# A walk on the wild side: *Oryza* species as source for rice abiotic stress tolerance

**DOI:** 10.1590/1678-4685-GMB-2016-0093

**Published:** 2017-03-20

**Authors:** Paloma Koprovski Menguer, Raul Antonio Sperotto, Felipe Klein Ricachenevsky

**Affiliations:** 1 Departamento de Botânica, Universidade Federal do Rio Grande do Sul (UFRGS), Porto Alegre, RS, Brazil; 2 Setor de Genética e Biologia Molecular do Museu de Ciências Naturais (MCN), Centro de Ciências Biológicas e da Saúde (CCBS), Programa de Pós-Graduação em Biotecnologia (PPGBiotec), Centro Universitário UNIVATES, Lajeado, RS, Brazil; 3 Programa de Pós-Graduação em Agrobiologia, Departamento de Biologia, Universidade Federal de Santa Maria (UFSM), Santa Maria, RS, Brazil

**Keywords:** Oryza, rice, wild species, abiotic stress, domestication

## Abstract

*Oryza sativa*, the common cultivated rice, is one of the most important crops for human consumption, but production is increasingly threatened by abiotic stresses. Although many efforts have resulted in breeding rice cultivars that are relatively tolerant to their local environments, climate changes and population increase are expected to soon call for new, fast generation of stress tolerant rice germplasm, and current within-species rice diversity might not be enough to overcome such needs. The *Oryza* genus contains other 23 wild species, with only *Oryza glaberrima* being also domesticated. Rice domestication was performed with a narrow genetic diversity, and the other *Oryza* species are a virtually untapped genetic resource for rice stress tolerance improvement. Here we review the origin of domesticated *Oryza sativa* from wild progenitors, the ecological and genomic diversity of the *Oryza* genus, and the stress tolerance variation observed for wild *Oryza* species, including the genetic basis underlying the tolerance mechanisms found. The summary provided here is important to indicate how we should move forward to unlock the full potential of these germplasms for rice improvement.

## Introduction

Continuous population and consumption growth are placing enormous demands on natural resources and agriculture. Today, approximately a billion people are chronically malnourished and it is uncertain whether our current agricultural system will be able to feed the expected world population, projected to reach nine billion by 2050. The efficient use of resources and reduced food waste can save much food, and increased crop production is fundamental to meet the world's future food needs (Godfray *et al.*, 2010; Foley *et al.*, 2011; Palmgren *et al.*, 2014). However, there is an urgent need to reduce agriculture's environment footprint, and farming land should not be expanded at the expense of natural ecosystems. We are faced with the challenge of increasing food production without degrading land, water and biodiversity in an environment becoming increasingly exposed to a myriad of abiotic stresses (Foley *et al.*, 2011; Mueller *et al.*, 2012; Sang and Ge, 2013; Palmgren *et al.*, 2014).

Rice is one of the world's most important staple crops, feeding more than 2.7 billion people worldwide (Muthayya *et al.*, 2014), and also a model for genomic research in monocots. It is cultivated on 150 million hectares of land, and its annual yield is close to 610 million tons (http://irri.org/). Due to global adverse climate changes, rice growth and productivity in recent years has been seriously affected by abiotic stresses such as cold, drought, heat, flood, and salt (Zhang *et al.*, 2015). Plants have evolved complex but not well understood responses. A complicated signaling network is effectively and timely initiated, which ultimately reprograms the expression of a large set of stress-responsive genes (Hong *et al.*, 2016), leading to a series of morphological, physiological, and biochemical changes (Scafaro *et al.*, 2011; Lei *et al.*, 2013). The acclimation processes that result from stress perception aims at protecting plants from damages and increases the chance of survival (Hu and Xiong, 2014). However, it is important to highlight that acclimation to stressful conditions does not occur in all plant species, and depending on stress intensity and duration, growth and yield can be severely affected. In nature, there is a wide variability to stress response and tolerance, making worthwhile the search for “tolerance genes” in wild relatives of cultivated species.

Abiotic stress is a major concern for rice production, and an increasing threat to food security considering climate change, population increase and area of arable land available. Drought, flooding and extreme temperatures should become more frequent according to predictions (Wang, 2005; Bita and Gerats, 2013; Hirabayashi *et al.*, 2013), which are likely to increase pressure on grain harvest. Food security is an issue to the human population as a whole, but especially in rural areas of Asia and Africa, which represent about 35% of the total rice harvest area. In Asia, where 700 million people live in extreme poverty, 30% of them are in regions that are prone to abiotic stresses such as flooding, drought and excess soil salinity (Ismail *et al.*, 2013). These stresses often occur in combination, and stress responsive pathways often show extensive cross-talk (Mittler 2006).

Flooding is a widespread environmental stress. The rapid decline in the oxygen (O_2_) diffusion rate during flooding is accompanied by a reduction in cellular O_2_ levels and an energy crisis, which are particularly severe when photosynthesis is limited or absent (Bailey-Serres and Voesenek, 2008). Although rice is considered a flood tolerant crop, only limited cultivars display tolerance to prolonged submergence (Niroula *et al.*, 2012), with most dying within 14 days of complete submergence.

Salinity is one of the most devastating abiotic stresses in rice, and the salt-affected soils currently account for about 20% of the total paddy rice planting area (Zhou *et al.*, 2016). Soil salinity has adverse effects on plant germination, strength, and yield (Munns and Tester, 2008). On exposure to salt stress, the ionic balance (especially Na^+^/K^+^ ratio) and distribution is the ultimate manifestation of several physiologic processes in response to salt stress (Chen Y *et al.*, 2013), creating an imbalance in the supplies of water and other nutrient solutes (Sengupta and Majumder, 2010). Most plants can adapt to low or moderate salinities, but their growth is severely limited above 200 mM NaCl. Therefore, plant survival and growth depends on adaptations to re-establish ionic homeostasis (Hasegawa *et al.*, 2000).

Cold stress is one of the major environmental factors limiting the growth, productivity, and geographical distribution of crops, mostly in temperate and high altitude areas, due to the tropical origin of the rice species (Cruz *et al.*, 2013; Zhang *et al.*, 2014). Low temperature can affect growth and development of rice plants during any developmental stage, from germination to grain filling. During germination, the most common symptoms of cold temperature damage are low percentage and delayed germination (Cruz *et al.*, 2013; Dametto *et al.*, 2015), resulting in yield decreases up to 25% of the final yield and in increased weed competition (Fujino *et al.*, 2004). During the vegetative stage, it can severely affect seedling establishment, leading to yellowing of the leaves, growth retardation, and decreased tillering (Cruz *et al.*, 2013). Low temperatures that occur at critical reproductive stages can adversely affect grain quality (incomplete grain maturation) or cause yield reductions (Jena *et al.*, 2012; Cruz *et al.*, 2013; Zhang *et al.*, 2014).

Availability of irrigation water is a limiting factor in attaining the full potential yield in many crops (Singh *et al.*, 2015). Drought is one of the most widespread and damaging environmental stress factors in plants (Krannich *et al.*, 2015), especially in rice, which is sensitive to drought stress because it is acclimated to either rain-fed or fully irrigated fields. The effect of drought may vary with the different genotypes, development stages, and degree and duration of drought stress (Wang *et al.*, 2011). Rice plants are highly sensitive to drought stress during vegetative stage (resulting in reduced height, tiller number, and leaf area), at the panicle initiation and booting stages (Jiang *et al.*, 2004; Wang *et al.*, 2011). In China, the average annual drought affected area is up to 27 million hectares and rice production has decreased by 70-80 billion Kg since the 1990s (Luo, 2010).

A decline in rice production caused by heat stress is one of the biggest concerns resulting from future climate change. Maximum and minimum daily temperatures, and the number of hot days and warm nights in a year, are estimated to increase over most land areas (IPCC, 2014). In addition, climate variability is predicted to increase, leading to frequent episodes of heat stress, often coinciding with key developmental stages in crops, such as flowering (Hirabayashi *et al.*, 2015). It is predicted that rice yields would be reduced by up to 10% with an average daily temperature increase of 1 °C (Peng *et al.*, 2004). During early growth stages of rice, the occurrence of heat stress inhibits seedling establishment, leading to non-uniform growth, and reduced yield. The physiological and genetic basis of the heat response during the seedling stage is poorly understood (Lei *et al.*, 2013). High temperatures at anthesis cause spikelet sterility due to the failure of anther dehiscence and the reduction in the number of germinating pollen grains on the stigma, leading to reductions in grain yield (Jagadish *et al.*, 2010).

Therefore, the development of cultivated rice with abiotic stress tolerance is needed to stabilize the production level of rice. The method currently practiced for improving abiotic stress tolerance in rice cultivars is to explore germplasm for desirable traits (Scafaro *et al.*, 2010). Recent attempts (until now limited to *O. sativa* species) have been successful, with backcrossing of *Oryza sativa* ssp. *japonica* and *indica* leading to substantial improvements in abiotic stress tolerance (Cheng *et al.*, 2007). As there are about 24 known species within the *Oryza* genus, a large source of genetic material remains virtually untapped. The search for variability in the ancestors of cultivated rice, which undergoes a long history of artificial selection, has great potential value for rice breeding. Stress tolerance traits are likely to be found in wild rice species (Tian *et al.*, 2015), which are recognized as an important genetic resource for cultivated rice improvement (Sakai and Itoh, 2010). In this review, we focus on the rice domestication history, the diversification of the *Oryza* genus, the mechanisms and underlying genetic basis for abiotic stress tolerance in species within the *Oryza* diversity, which illustrates the feasibility of using rice wild relatives as a source for breeding stress tolerant *O. sativa*.

## Diversity in the *Oryza* genus

Rice belongs to the genus *Oryza*, of the tribe Oryzeae, subfamily Ehrhartoideae and family Poaceae (Grass Phylogeny Working Group, 2001). The tribe Oryzeae includes nine or ten genera and up to 70 species, and is very likely monophyletic; in this tribe, *Oryza* L. and *Leersia* Sw. are the two largest genera (reviewed by Kellogg, 2009). The analysis of both nuclear and chloroplast genes revealed that *Oryza* and *Leersia* are sister genera. The divergence of *Oryza–Leersia* from the other genera occurred approximately 20.5 MYA and the divergence of *Oryza* from *Leersia* 14.2 MYA (Ge *et al.*, 2002; Guo and Ge, 2005).

The *Oryza* genus consists of 24 species spread worldwide (Table 1) (Kellogg, 2009; Jacquemin *et al.*, 2013). The phylogeny of the *Oryza* genus spans approximately 15 million years (MY) of evolutionary history, a process that created diverse ecological adaptations (Vaughan *et al.*, 2003; Ammiraju *et al.*, 2010). The *Oryza* species have 11 different genome types (AA, BB, CC, BBCC, CCDD, EE, FF, GG, KKLL, HHJJ, and HHKK), and a 3.6-genome size variation (Lu *et al.*, 2009; Jacquemin *et al.*, 2013; Atwell *et al.*, 2014).

**Table 1:** *Oryza* species, including their genome types, life span, geographical distributions, habitats and available accessions.

Species	Genome^a^/Life history^b^	Distribution^c^	Usual habitat^d^	Accessions^e^
*Oryza alta*	CCDD / Perennial	Belize, Brazil, Colombia, Guyana and Paraguay	Savanna and sometimes in woodland; wet sites; open	27
*Oryza australiensis*	EE / Perennial	Northern Australia	Undulating plains of *Eucalyptus* and *Leptochloa* or box woodland; wet sites; open	52
*Oryza barthii*	AA / Annual	Benin, Botswana, Burkina Faso, Cameroon, Central Africa Republic, Chad, Côte d’Ivoire, Ethiopia, The Gambia, Ghana, Guinea, Mali, Mauritania, Namibia, Niger, Nigeria, Senegal, Sierra Leone, Sudan, Tanzania, and Zambia	Mopane or savanna woodland, savanna or fadama; deep water; open	405
*Oryza brachyantha*	FF / Annual	Burkina Faso, Cameroon, Central African Republic, Chad, Democratic Republic of Congo, Guinea, Mali, Niger, Senegal, Sierra Leone, Sudan, Tanzania, and Zambia	Flat ironstone rocks, granite/lateritic outcrops; water up to 0.5 m deep, but more often in shallow water; open	36
*Oryza coarctata*	KKLL / Perennial	India, Sri Lanka, Bangladesh and Myanmar	Salt marsh ecosystems; inundated (twice a day) with saline river or seawater; open	6^f^
*Oryza eichingeri*	CC / Perennial	Central Africa Republic, Côte d’Ivoire Democratic Republic of Congo, Kenya, Rwanda, Sri Lanka, Tanzania, and Uganda	Undisturbed forest, gallery or evergreen forest, or forest margins; damp or flooded sites; shade or semi-shade	37
*Oryza glaberrima*	AA / Annual	West Africa	Upland to deep water; open	9073
*Oryza glumaepatula*	AA / Perennial	Bolivia, Brazil, Colombia, Costa Rica, Cuba, Dominican Republic, French Guiana, Guyana, Honduras, Mexico, Panama, Surinam, and Venezuela	Swamps and marshes; usually deep water; open	76
*Oryza grandiglumis*	CCDD / Perennial	Argentina, Bolivia, Brazil, Colombia, Ecuador, French Guiana, Paraguay, and Peru	Savanna or woodland; water at river's edges or wet sites; open or shade	16
*Oryza granulata*	GG / Perennial	India, Cambodian, Vietnam, Thailand, southern China, Malaysia, Philippine, Nepal and Sri Lanka	Forest floor; damp sites; shade	29
*Oryza latifolia*	CCDD / Perennial	Argentina, Belize, Bolivia, Brazil, Colombia, Costa Rica, Cuba, Dominican Republic, Ecuador, El Salvador, French Guiana, Guatemala, Guyana, Haiti, Honduras, Mexico, Nicaragua, Panama, Paraguay, Peru, Puerto Rico, Surinam, Trinidad and Venezuela	Low forest, rainforest, secondary growth forest, open woodland, undulating savanna, pasture, cultivated fields, open swamp, hill slopes, high ridges, coastal belts; wet or damp sites; open or semi-shade	86
*Oryza longiglumis*	HHJJ / Perennial	Indonesia and Papua New Guinea	Forest areas; seasonally wet; shade or semi-shade	6
*Oryza longistaminata*	AA / Perennial	Angola, Benin, Botswana, Burkina Faso, Burundi, Cameroon, Central Africa Republic, Chad, Congo, Côte d’Ivoire, Democratic Republic of Congo, Ethiopia, Gabon, The Gambia, Ghana, Kenya, Madagascar, Malawi, Mali, Martinique, Mozambique, Namibia, Niger, Nigeria, Rwanda, Senegal, Seychelles, Sierra Leone, Somalia, South Africa, Sudan, Tanzania, Uganda, Zambia, and Zimbabwe	Savanna or openings in rain or gallery forests; deep water; open	309
*Oryza malampuzhaensis*	BBCC / Perennial	Western Ghats of South India	Forest pools; seasonally dry; shade	12
*Oryza meridionalis*	AA / Annual	Australia, Indonesia, and Papua New Guinea	Edges of seasonally freshwater lagoons, temporary pools, and swamps; seasonally wet; open	65
*Oryza meyeriana*	GG / Perennial	Indonesia, Malaysia, Philippines, and Thailand	Forest areas; damp sites; shade	25
*Oryza minuta*	BBCC / Perennial	Philippines and Papua New Guinea	Lowland areas beside streams and riverbanks; seasonally wet; shade or semi-shade	86
*Oryza nivara*	AA / Annual	Bangladesh, Cambodia, China, India, Laos, Malaysia, Myanmar, Nepal, Sri Lanka, Thailand, and Vietnam	Swampy areas; seasonally wet and shallow water; open	2064
*Oryza officinalis*	CC / Perennial	Australia, Bangladesh; Brunei, Cambodia, China, India, Indonesia, Laos, Malaysia, Myanmar, Nepal, Papua New Guinea, Philippines, Thailand, and Vietnam	Edge of or in forests, open vegetation, in abandoned cultivated rice fields, in Southeast Asia near the coast; wet sites; open or semi-shade	317
*Oryza punctata*	BB / Annual	Angola, Benin, Cameroon, Central Africa Republic, Chad, Congo, Côte d’Ivoire, Democratic Republic of Congo, Ethiopia, Ghana, Kenya, Madagascar, Malawi, Mozambique, Nigeria, Sudan, Swaziland, Tanzania, Uganda, Zambia, and Zimbabwe	Seasonally swamp areas, around water holes and pools, on riverbanks in areas that flood to 1 m depth; open	98^g^
*Oryza punctata*	BBCC / Perennial	Angola, Benin, Cameroon, Central Africa Republic, Chad, Congo, Côte d’Ivoire, Democratic Republic of Congo, Ethiopia, Ghana, Kenya, Madagascar, Malawi, Mozambique, Nigeria, Sudan, Swaziland, Tanzania, Uganda, Zambia, and Zimbabwe	Forest areas; wet sites; shade	98^g^
				
*Oryza rhizomatis*	CC / Perennial	Sri Lanka	Tropical forest and open, tall scrub with grassy openings; swampy or periodically flooded areas; open or semi-shade	21
*Oryza ridleyi*	HHJJ / Perennial	Cambodia, Indonesia, Laos, Malaysia, Myanmar, Papua New Guinea, and Thailand	Marshes or near streamsides in forest; seasonally wet; shade	21
*Oryza rufipogon*	AA / Perennial	Australia, Bangladesh, China, India, Indonesia, Laos, Malaysia, Myanmar, Nepal, Papua New Guinea, Philippines, Sri Lanka, Thailand, and Vietnam	Swamps and marshes; seasonally deepwater and wet year round; open	1617
*Oryza sativa*	AA / Annual	Southeast Asia	Upland to deep water; open	212982
*Oryza schlechteri*	HHKK / Perennial	Indonesia and Papua New Guinea	Forest areas; wet sites; shade or semi-shade	3

According to ^a^ Jacquemin *et al.*, 2013, ^a,b,c,d^ Lu and Jackson 2004, ^a,b,c,d^ Sengupta and Majumder 2010, ^a,c,d^ Vaughan *et al.*, 2003

e Number of accession entries per species in the Genesys Database (http://www.genesys-pgr.org/).

f Includes accessions registered as Oryza/Porteresia coarctata.

g Includes accessions from both annual and perennial *O. punctata*.

A phylogenomic study sampled and sequenced 142 single-copy genes to clarify the relationships among all diploid genome types of the rice genus (Zou *et al.*, 2008). The analysis identified two episodes of rapid speciation that occurred approximately five and ten million years ago (MYA) and gave rise to almost the entire diversity of the genus. The first event occurred approximately ten MYA (Guo and Ge, 2005) and led to a rapid diversification of the G genome, F genome and a lineage that subsequently diversified into the rest of the rice genomes. Additionally, the H, J and K genomes that are now only present in tetraploid species also diverged around this time (Ge *et al.*, 1999; Guo and Ge, 2005). The second event led to the diversification of the A, B, and C genomes approximately five MYA, confirming that the A and B genome species are sisters and the C genome clade is sister to that (Zou *et al.*, 2008, Kellogg, 2009).

Divergence of the A genome took place over the past two million years, and the current most divergent species within this group are the perennial *O. meridionalis* (rhizomatous) and *O. longistaminata* (Zhu and Ge, 2005; Vaughan *et al.*, 2008). *O. sativa* has two presumed wild ancestors (*O. rufipogon* and *O. nivara*), while the annual *O. barthii* is the progenitor of African domesticated rice *O. glaberrima* (please see next section). The two cultivated species and their respective progenitors shared an unknown ancestral of about 0.86 MYA (Sarla and Swamy, 2005; Zhu *et al.*, 2014). Perennial *O. glumaepatula* is the only A genome group member with a current distribution in Latin America and presents a strong relation between genetic and geographic distances (Jacquemin *et al.*, 2013; Vaughan *et al.*, 2003). The A genome species *O. meridionalis, O. longistaminata, O. glaberrima and O. barthii* are considered candidates for tolerance to heat and drought stresses based on their distribution in temperature and moisture extremes (Atwell *et al.*, 2014).

The CC genome group is formed by *O. officinalis, O. rhizomatis* and *O. eichingeri*. All three species are perennial, occurring in shade or semi-shade forest environments, among other habitats. *O. officinalis* (generally rhizomatous) has a high level of genetic diversity between populations, and *O. rhizomatis* (rhizomatous) is endemic to Sri Lanka (Vaughan *et al.*, 2003; Lu and Jackson, 2004). The only member of the diploid BB genome group is *O. punctata*, which also exists in the allotetraploid form BBCC. Both BB and BBCC populations are distributed in Africa but occupying distinct niches. The diploid form is found in open habitats, while the allotetraploid in shade or semi-shade environments (Vaughan *et al.*, 2003). Both forms of *O. punctata* are potential genetic reservoirs for tolerance to drought stress if considering their plasticity over moisture extremes (Atwell *et al.*, 2014). *O. eichingeri* is believed to be the CC genome donor to the allotetraploid *O. punctata*, while the diploid *O. punctata* is the BB genome donor to the other allotetraploid BBCC species *O. minuta* and *O. malampuzhaensis* (rhizomatous). Perennial *O. minuta* and *O. malampuzhaensis* seem to have arisen from different polyploidy events if considering their distinct morphology, distribution and genetic diversity (Jacquemin *et al.*, 2013; Vaughan *et al.*, 2003). *O. minuta* diploid progenitors are *O. punctata* (BB), as already mentioned, and *O. officinalis* (CC), and its recent polyploidization is believed to have occurred within the last 400,000 years (Lu *et al.*, 2009). *O. malampuzhaensis*, endemic to the Nallamalais of Eastern Ghats (India), is under severe threat considering its narrow distribution over a small geographical area, which leads to vulnerability to habitat destruction and fragmentation (Elangovan *et al.*, 2012). The CCDD genome species *O. latifolia, O. alta* and *O. grandiglumis* are very closely related, with current distribution in Latin America. In fact, some studies suggest that CCDD group members are one complex species with different ecotypes (Vaughan *et al.*, 2003). *O. alta* polyploidization was estimated to have happened less than 1.6 MYA. Additionally, *O. grandiglumis* and *O. latifolia* are considered candidates for tolerance to flooding stress (Lu *et al.*, 2009; Atwell *et al.*, 2014).

The GG genome species *O. granulata* and *O. meyeriana* are found in forest shade environments. *O. granulata* occupies the most basal position in the *Oryza* genus phylogeny, presents a high level of genetic diversity between populations, and is a potential genetic resource for tolerance to cold stress (Aggarwal *et al.*, 1997; Vaughan *et al.*, 2003; Atwell *et al.*, 2014). The F genome species *O. brachyantha* has a compact genome (the smallest in the *Oryza* genus) and is distributed in West and East Africa (Chen J *et al.*, 2013). Limited information is available for *O. coarctata* (KKLL), *O. longiglumis* (HHJJ), *O. ridleyi* (HHJJ) and *O. schlechteri* (HHKK) (Vaughan *et al.*, 2003; Jacquemin *et al.*, 2013). Regarding abiotic stresses, *O. coarctata* shows considerable adaptation to salinity, and *O. ridleyi* and *O. schlechteri* are considered good genetic reservoirs for tolerance to flooding (Lu *et al.*, 2009; Sengupta and Majumder, 2010; Atwell *et al.*, 2014). A summary of all *Oryza* species cited in this review, including their genotypes, life span, geographical distributions and habitats, is presented in Table 1.

## The origin of Asian cultivated rice

There are two distinct groups of Asian cultivated rice, namely *O. sativa* ssp. *japonica* and *indica*, which can be differentiated by morphological and physiological traits, additionally to an incomplete sterility barrier (Sang and Ge, 2013). The origin and evolution of the *japonica* and *indica* subspecies is under considerable debate over the past several years. Two models of interactive domestication scenarios were proposed. The ‘*snowballing model*’ suggests a single domestication event that created an early cultivar with a set of domestication traits. This early cultivar, when hybridized with different wild rice populations, *japonica*-like and *indica*-like, would have enabled the fixation of critical domestication alleles in each one of them separately. Alternatively, in the ‘*combination model*’, both subspecies were domesticated independently from diverse wild rice ecotypes with subsequent hybridization, leading to the introgression and fixation of domestication alleles (Sang and Ge, 2007).

Considering archeological and genetic studies available to date, the ‘*combination model*’ is suggested as the closest scenario from what had happened during Asian rice domestication (Gross and Zhao, 2014). Briefly, the *japonica* subspecies was originated from *O. rufipogon* in the Yangtze River Valley in China, where rice cultivation possibly started around 8,000 years ago. Meanwhile, *indica* or *proto-indica* independent origin of cultivation probably took place in the Ganges plains in India, but *proto-indica* domestication was only complete after domesticated *japonica* was introduced and hybridized with *indica*, around 4,000 years ago (Fuller *et al.*, 2009, 2011; Gross and Zhao, 2014). Both *O. rufipogon* and its annual derived *O. nivara* are native to India today. In most of the physiological and morphological traits, *O. nivara* is rather similar to the cultivated rice than *O. rufipogon*. Thus, *O. nivara* could have served as the *indica* progenitor, and domestication would have required fewer genetic modifications or mating system transitions (Li *et al.*, 2006; Sang and Ge, 2013). If this hypothesis is correct, selection would have been less intense for *indica* because *O. nivara* already presented several cultivated rice traits. Thus, key domestication alleles had more chance to arise during *japonica* domestication from *O. rufipogon*, and semi-domesticated *indica* became the primary recipient of the domestication alleles when *japonica* was brought to India (Vaughan *et al.*, 2008; Sang and Ge, 2013).

During the process of crop domestication many of the inherited traits involved in biotic and abiotic stress resistance may have been weakened or lost, since it is estimated that only 10-20% of wild species diversity is present in cultivated rice (Zhu *et al.*, 2007; Palmgren *et al.*, 2014). One of the most important resources for improvement of cultivated rice is the genetic reservoir hidden in wild rice species distributed across several biomes worldwide. It is also important to underline that isolated populations within species may also contain critical genes (Jacquemin *et al.*, 2013; Atwell *et al.*, 2014). Rice gene banks around the world exhibit an extensive seed collection, covering the genetic diversity present in farmers’ cultivars, landraces and Oryza species. The two largest gene banks are the International Rice Research Institute in the Philippines (4,370 wild species and hybrids accessions at IRRI, http://irri.org), and Oryzabase in Japan (1,703 entries, http://www.shigen.nig.ac.jp) (Jacquemin *et al.*, 2013). Moreover, the Genesys database (http://www.genesys-pgr.or) allows searching accessions for many species in several seed banks, being a valuable resource for germplasm distribution (see total number of accessions available for each Oryza species in Table 1). A genus-wide comparative genome platform is essential to understand the genetic differences associated with abiotic factors. The sequencing of 16 *Oryza* genomes is either complete or underway with “gold standard” reference sequences available for the cultivated species *O. sativa* ssp. *japonica* and *O. glaberrima*, and for the wild species *O. barthii* and *O. brachyantha* (Jacquemin *et al.*, 2013). The introgression of desirable traits via conventional breeding into cultivated rice should be more feasible from close relatives (although a viable hybrid *O. coarctata* X *O. sativa* has been reported – see below). Among the AA genome species, introgression lines can be obtained by backcrossing F1 hybrids that are partially sterile, and various levels of postzygotic barriers are known (Doi *et al.*, 2008). The gene groups that cause these incompatibilities were genetically identified, but further studies are needed to break the reproductive isolation (Ji *et al.*, 2005; Qiu *et al.*, 2005). When *O. sativa* is crossed to non-AA genome species, viable but highly sterile F1 hybrids can be obtained using embryo culture. However, the aberrant chromosome pairing in meiosis makes it difficult to introgress genetic information from these species into cultivated rice (Doi *et al.*, 2008). Transgenic approaches will be eventually needed for genes from taxonomically distant species (Vaughan *et al.*, 2003; Palmgren *et al.*, 2014). Therefore, it is of great significance to understand the genetic diversity within the wild relatives of *Oryza* in the context of their natural environment of origin, in order to identify the genetic basis of phenotypic variation between and within-species. This knowledge is an important resource to improve rice production under abiotic stresses (Sang and Ge, 2013; Gross and Zhao, 2014; Palmgren *et al.*, 2014).

## Abiotic stress tolerance in wild relatives: submergence

Rice is commonly grown in flooded soil containing a thin water layer. Although rice roots are well adapted to hypoxic conditions, most rice cultivars die rapidly if their shoots are submerged (Mackill *et al.*, 2012). Several rainfed rice areas are at risk of flooding, a stress that decreases yield substantially (Ismail *et al.*, 2013). Submergence inhibits aerobic metabolism and photosynthesis, leading to carbohydrate depletion and, depending on the stress intensity, plant death (Fukao and Bailey-Serres, 2004). However, rice plants are able to tolerate submergence using different strategies. In the low oxygen escape syndrome (or “escape strategy”) shoot elongation is stimulated by progressive flooding, keeping leaves in contact with the air and escaping the increasing levels of water (Bailey-Serres and Voesenek, 2008). In the “quiescent strategy”, hypoxia reduces elongation, represses carbohydrate degradation and stimulates anaerobic metabolism. When water recedes, plants resume growth. While the escape strategy only results in submergence tolerance if flooding occurs progressively, rice genotypes that use the quiescent strategy are able to survive up to 14 days of complete submergence (Bailey-Serres and Voesenek, 2008). Both strategies were elucidated at the molecular level: the quiescent strategy is linked to the SUB1 locus, while the escape strategy is dependent on the SNORKEL (SK) locus (for reviews see Bailey-Serres and Voesenek, 2008; Mickelbart *et al.*, 2015).

**Figure 1:**
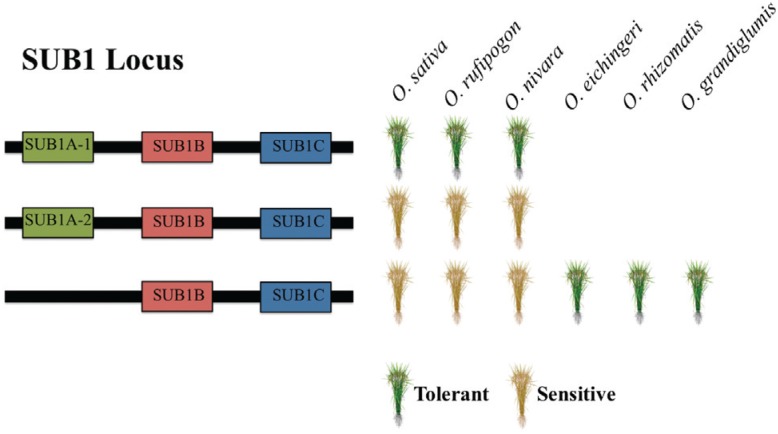
Submergence tolerance and genetic architecture of SUB1 locus in cultivated and wild *Oryza* species. Genotypes from AA genome species *Oryza sativa, Oryza rufipogon* and *Oryza nivara* are tolerant or sensitive to submergence depending on the presence of SUB1A-1 allele of the SUB1A gene. Genotypes that either have SUB1A-2 allele or lack a SUB1A, carrying only SUB1B and SUB1C genes, are sensitive. Genotypes from CC genome species *Oryza eichingeri, Oryza rhizomatis* and the CCDD tetraploid species *Oryza grandiglumis* were shown to be tolerant to submergence while carrying a SUB1A gene-lacking SUB1 locus, indicating the locus does not contribute to the stress tolerance in these species.

SUB1 is the major QTL associated with submergence tolerance in rice cultivars that use the quiescent strategy (Fukao *et al.*, 2009). The SUB1 locus was mapped to chromosome 9, and is composed of a cluster of ethylene response factors (ERF) genes located *in tandem*, named *SUB1A, SUB1B* and *SUB1C* (Figure 1). Different rice genotypes have two genes in the cluster, *SUB1B* and *SUB1C*, whereas a third gene, *SUB1A*, is present only in a subset of them. Interestingly, tolerance to submergence is linked to a specific allele of the *SUB1A* gene, named *SUB1A-1* (Xu *et al.*, 2006). Accessions that lack *SUB1A* gene, or carry the *SUB1A-2* allele, are sensitive to submergence (Figure 1). Introgression of functional copies of *SUB1A-1* in sensitive genotypes is sufficient to generate tolerant plants (Xu *et al.*, 2006).

The tolerant *SUB1A-1* allele is derived from the *aus* subgroup of *indica* rice (Xu *et al.*, 2006). Wild species from the *Oryza* genus commonly grow in constantly or seasonally wet habitats (Vaughan *et al.*, 2003), and thus submergence tolerance could be found in other species. Niroula *et al.* (2012) tested 109 accessions of rice and wild relatives, including 12 species, for submergence tolerance, and found *O. rufipogon* and *O. nivara* (AA genome; Table 2) tolerant accessions that carry the *SUB1A-1* allele, showing that SUB1 locus architecture determines submergence tolerance in these species, as in *O. sativa* (Figure 1; Niroula *et al.*, 2012). Strikingly, accessions of *O. rhizomatis* and *O. eichingeri* (CC genome; Table 2) were also found to be submergence tolerant, but *SUB1A* sequences were absent from genomes of tested accessions, indicating that a novel, SUB1A-independent mechanism is responsible for submergence tolerance, at least in these two CC genome species (Figure 1; Niroula *et al.*, 2012).

**Table 2:** Tolerance to abiotic stresses found in accessions of *Oryza* species and references.

Species	Tolerance compared to *O. sativa*	Reference
*Oryza coarctata*	Salt	Sengupta and Majumder, 2009
*Oryza eichingeri*	Submergence	Niroula *et al*., 2012
*Oryza glaberrima*	Salt, Drought	Ndjiondjop *et al.,* 2010, Platten *et al*., 2013
*Oryza glumaepatula*	Submergence	Hattori *et al*., 2009
*Oryza grandiglumis*	Submergence	Okishio *et al*., 2014, 2015
*Oryza meridionalis*	Heat	Scafaro *et al*., 2010
*Oryza nivara*	Drought	Singh *et al*., 2015
*Oryza officinalis*	Drought, Heat (Early Morning Flowering)	Ishimaru *et al*., 2010, Feng *et al*., 2012
*Oryza rhizomatis*	Submergence	Niroula *et al*., 2012
*Oryza rufipogon*	Salinity, Cold, Drought, Submergence	Hattori *et al*., 2009, Tian *et al*., 2011, Xiao *et al*., 2014

*Oryza grandiglumis* is a tetraploid species with CCDD genome (Table 2) that grows in Amazonian floodplains, where water levels can reach up to 10 meters, and thus it was expected to show some degree of submergence tolerance (Okishio *et al.*, 2014, 2015). Depending on the flooding conditions, *O. grandiglumis* showed distinct responses: when progressively submerged, the internodes elongated, resembling the escape strategy of *O. sativa*; however, when plants were completely submerged, growth was reduced, as in the quiescent strategy (Okishio *et al.*, 2014). Interestingly, SUB1A is absent in *O. grandiglumis*, indicating that a SUB1A-independent mechanism for a quiescent strategy is present, as observed for *O. rhizomatis* and *O. eichingeri* (Figure 1). Genes similar to *SNORKEL1* and *SNORKEL2*, responsible for the escape strategy in deepwater-adapted *O. sativa*, as well as *O. rufipogon* and *O. glumeapatula* (AA genome; Table 2), are absent in *O. grandiglumis* (Hattori *et al.*, 2009, Okishio *et al.*, 2015). These results indicate that *O. grandiglumis* is tolerant to both gradual and full submergence by unknown mechanisms (Okishio *et al.*, 2014), indicating that CC genome *Oryza* species might provide new molecular mechanisms to improve cultivated rice.

## Salinity

Soil salinization is a worldwide problem for agriculture. It affects 6% of total Earth's land, as a result of natural accumulation over long periods of time (Rengasamy, 2002). However, agricultural activity contributes to secondary salinization: 2% of all dry land is becoming salinized, and more than 20% of irrigated soils are affected, mostly because of irrigation water containing small amounts of sodium chloride (Tester and Davenport, 2003).

Plants vary in their sensitivity to salt stress, and rice is the most sensitive among cereals. Salinity reduces growth rate, including cellular and leaf expansion, number of tillers and photosynthesis, and can lead to premature senescence of older leaves (Munns and Tester, 2008, Sirault *et al.*, 2009). The deleterious effects of salt in plants can be a result of osmotic stress (caused by salt in the soil), or of ionic stress (toxic effect of Na^+^ accumulation in plant tissues; Munns and Tester, 2008). The tolerance to ionic stress is dealt with by Na^+^ exclusion from xylem vessels to avoid shoot accumulation, or by tissue tolerance, when Na^+^ levels reach toxic levels and plant leaf cells compartmentalize salt to reduce damage (Roy *et al.*, 2014).

The genetic basis of tolerance to ionic stress is much better understood than to osmotic stress (Roy *et al.*, 2014). A range of transporters involved in reducing Na^+^ accumulation in shoots and in subcellular compartmentalization was described, such as the high affinity potassium transporter (HKT), salt overly sensitive (SOS) and Na^+^/H^+^ exchanger (NHX) gene families (Mickelbart *et al.*, 2015). HKT members are crucial determinants of tissue concentration of Na^+^. *OsHKT1;5* is the causative gene of *Saltol*, the major quantitative trait locus (QTL) for salt accumulation in *O. sativa* genotypes (Ren *et al.*, 2005). OsHKT1;5 is a plasma membrane transporter that regulates partitioning of Na^+^ between roots and shoots by efflux of Na^+^ from the xylem to adjacent parenchyma cells (Hauser and Horie, 2010). Four amino acid changes in OsHKT1;5 resulted in increased Na^+^ efflux activity in salt tolerant *indica* cultivar Nona Bokra compared to the salt sensitive cultivar Koshihikari (Ren *et al.*, 2005). Although other QTLs were described (Negrão *et al.*, 2011), *Saltol* is the only one that has been cloned so far.

In a large screening effort which included several *O. sativa* cultivars, landraces and *O. glaberrima* (AA genome, Table 2) genotypes, it was shown that salinity sensitivity is correlated with Na^+^ concentration in leaf blades. OsHKT1;5 genotype was shown to be a major determinant for tolerance: the more active the efflux transporter, which directs the Na^+^ exclusion from the transpiration stream, the less Na^+^ is translocated to leaves (Platten *et al.*, 2013). Interestingly, these authors found tolerant *O. glaberrima* accessions with low Na^+^ concentration in leaves, but carrying *OsHKT1;5* alleles that are associated with salt sensitivity and Na^+^ accumulation in leaves of *O. sativa* genotypes. These results indicate that *O. glaberrima* genotypes could exclude Na^+^ from shoots using a mechanism independent of OsHKT1;5 (Platten *et al.*, 2013).

Other species from the genus *Oryza* were explored for salt tolerance genes. *O. rufipogon* was shown to be salt tolerant when compared to rice sensitive cultivars (Zhou *et al.*, 2016). Introgression lines derived from *O. rufipogon* X *O. sativa* cross revealed 15 QTLs for salinity tolerance, 13 of them derived from the *O. rufipogon* parent (Tian *et al.*, 2011). Over-expression of bHLH transcription factors *OrbHLH001* and *OrbHLH2* from *O. rufipogon* resulted in Arabidopsis and *O. sativa* salt tolerant lines (Zhou *et al.*, 2009; Li *et al.*, 2010; Chen Y *et al.*, 2013). These authors showed that OrbHLH001 is able to positively regulate the K^+^ transporter OsAKT1, suggesting that salt tolerance results from maintenance of K^+^ homeostasis under high Na^+^ conditions (Chen Y *et al.*, 2013). Based on heterologous expression in Arabidopsis, OrbHLH2 was suggested to positively regulate genes from the CBF/DREB pathway (Zhou *et al.*, 2009). Indeed, transcriptomic studies of the *O. rufipogon* response to high salinity showed that transcription factors are among the top up-regulated genes (Zhou *et al.*, 2016).

## *Oryza coarctata*, a promising source of salinity and submergence genes for rice

*Oryza coarctata* (also known as *Porteresia coarctata*, Lu and Ge, 2003) is an allotetraploid wild rice with extreme salt and submergence tolerance. It is unique among wild rice species, since it has a KKLL genome (Table 1; Lu *et al.*, 2009). *O. coarctata* grows in coastal region of India and Bangladesh, where it experiences lunar tides and is submerged with saline seawater every 12 hours (Sengupta and Majumder, 2010, Garg *et al.*, 2014). It has also been established as an important resource for prospecting genes to improve cultivated rice (Garg *et al.*, 2014). A hybrid derived from a cross between *O. sativa* and *O. coarctata* has been reported (Jena, 1994), and salt tolerant rice cultivars with introgressed *O. coarctata* traits for salt tolerance are currently under development in the International Rice Research Institute (IRRI; www.irri.org). Still, little is known about the physiological and molecular details of this wild rice adaptation to high salinity conditions.

*O. coarctata* growth, relative water content and photosynthesis are unaffected by high concentrations (400 mM) of NaCl, conditions in which *O. sativa* salt tolerant cultivars do not develop properly (Sengupta and Majumder, 2009). Leaves of *O. coarctata* contain “salt hairs”, outgrowths of the epidermis that increase their number under high salinity and secrete excessive salt. When salt concentration in the growth media is high (300-400 mM), the salt hairs in the abaxial surface collapse and fall off from the leaf surface (Sengupta and Majumder, 2009). Still, total Na^+^ concentration in leaves of *O. coarctata* does not increase under salt stress, indicating that *O. coarctata* avoids Na^+^ toxicity in mesophyll cells by compartmentalization of salt in epidermal hairs, a mechanism similar to what is known for other halophyte grasses (Sengupta and Majumder, 2009, 2010). The secreted salt is a significant proportion of the Na^+^ reaching leaves, and important to maintain a low Na:K ratio (Sengupta and Majumder, 2010). A tonoplast-localized transporter from the NHX family of *O. coarctata* was recently cloned. *PcNHX1* transcription is regulated during the day, presumably reflecting the circadian variation in tide experienced by the plant, and is also rapidly induced by NaCl treatment, compartmentalizing Na^+^ into vacuoles (Kizhakkedath *et al.*, 2015). Thus, *O. coarctata* is adapted to high salinity environments by using multiple mechanisms to cope with salt stress, including decreased root-to-shoot translocation and increased compartmentalization in the vacuole and secretion in salt hairs.

Proteomic analyses identified up-regulated proteins by Na^+^ treatment in *O. coarctata*, including transcription factors of the CBF/DREB pathway of abiotic stress response (Shinozaki and Yamaguchi-Shinozaki, 2000); a cellulose synthase-like, which could help maintaining cellulose synthesis during salt stress (Endler *et al.*, 2015); and an L-myo-inositol 1-phosphate synthase, important for inositol synthesis. Inositol metabolism is, in fact, one of the most studied aspects of *O. coarctata* salt tolerance. Its derivative, pinitol, is a known osmoprotectant in many plant species, and accumulates in *O. coarctata* under high salinity (Sengupta *et al.*, 2008; Sengupta and Majumder, 2010). Strikingly, cloning and characterization of *O. coarctata* L-myo-inositol 1-phosphate synthase (*INO1*) showed that enzyme activity is maintained properly even in high salt concentrations, and that its expression in plants and bacteria confers high, albeit variable, salt tolerance to these organisms (Majee *et al.*, 2004; Das-Chatterjee *et al.*, 2006; Sengupta and Majumder, 2010). This highlights the potential of wild species to provide useful proteins for rice (and other crops) improvement. Moreover, inositol methyl transferase (IMT1), which methylates inositol into pinitol, is up-regulated in the same conditions as INO1 in *O. coarctata*, indicating that inositol synthesis and conversion to pinitol are key steps for salt tolerance in this species (Sengupta *et al.*, 2008).

More recently, a study evaluated *O. coarctata* transcriptomic changes under salt and submergence stresses (alone or combined, compared to control conditions), and found several transcription factors up-regulated in leaves under stress conditions, such as *NAC, WRKY* and *MYB* gene family members, indicating extensive transcriptional regulation in stress responses (Garg *et al.*, 2014). Gene Ontology analyses showed enrichment of ABA-responsive genes under salinity stress, and of carbohydrate metabolism and anaerobic respiration genes under submergence stress. In plants under submergence stress, genes related to ethylene and gibberellin responses were also identified, along with Alcohol Dehydrogenase, a marker for anoxia stress, indicating that a SUB1A-related response might be present in *O. coarctata* (Garg *et al.*, 2014; see above for discussion on submergence stress mechanisms). However, demonstration of the presence of SUB1A-like ERF transcription factors and of their role in submergence response in *O. coarctata* is lacking. Moreover, suberin and cellulose synthesis-related transcripts were identified as up-regulated in both stresses, indicating that these processes might be key for stress tolerance, as observed in other species (Garg *et al.*, 2014; Endler *et al.*, 2015; Barberon *et al.*, 2016).

## Cold

The *japonica* cultivars of *O. sativa* are usually adapted to temperate climates, a process that was driven by domestication, while *indica* cultivars are generally tropical (Kovach *et al.*, 2007; Ma *et al.*, 2015). Thus, temperate *japonica* cultivars are more tolerant to low temperatures than *indica*, and introgression of cold tolerance traits from temperate into tropical genotypes is desirable (Cruz *et al.*, 2013; Dametto *et al.*, 2015). However, little is known about the molecular basis for low temperature tolerance in *O. sativa*, and even less about the variation of cold tolerance among its wild relatives.

The *Oryza* genus has a pan-tropical distribution, growing in regions with an average low temperature of 15 °C or above during the growth season, with only a few exceptions (Atwell *et al.*, 2014). Hence, it is possible that other species of *Oryza* might be better adapted to lower temperatures, being able to provide alleles to improve cold tolerance. Atwell *et al.* (2014) used the distribution of each species in different climates to estimate the best candidates for stress tolerance, and *O. granulata* is suggested as a possible source for cold tolerance. *O. eichingeri* (CC genome, Table 2) also grows in low temperature environments, and could be considered a good candidate (Atwell *et al.*, 2014). However, screenings of cold tolerance are still lacking for most *Oryza* species.

One genotype of *O. rufipogon*, named Dongxiang wild rice, is able to withstand overwintering in its natural habitat and temperature as low as 3 °C for three days in laboratory conditions (Xiao *et al.*, 2014; Mao *et al.*, 2015). Using an experimental population derived from Dongxiang wild rice X Nanjing 11 (a cold sensitive cultivar) crosses, a *CBF3/DREB1G* gene was found to co-localize to a previously identified cold-related QTL (Xiao *et al.*, 2014). CBF/DREBs are known regulators of cold and other abiotic stress responses (Mao and Chen, 2012; Mickelbart *et al.*, 2015). Interestingly, *CBF3/DREB1G* is up-regulated as early as three hours after cold treatment in both Dongxiang wild rice, but only after 12 hours in the sensitive one. Genes known to be downstream of CBF/DREB1 in the cold response are up-regulated accordingly in both tolerant genotypes (Xiao *et al.*, 2014). Other QTLs unique to Dongxiang wild rice were described (Mao *et al.*, 2015).

Recently, a SNP associated with temperate *japonica* cold tolerance was described. COLD1 (*chilling-tolerance divergence*) is a plasma membrane- and endoplasmic reticulum-localized regulator of G protein that activates Ca^2+^ influx during cold sensing. One SNP that results in an amino acid change was found to be responsible for the difference in cold tolerance. The SNP found in *japonica* is shared with accessions of *O. rufipogo*n, but not with *O. nivara* or *O. barthii* (AA genome; Ma *et al.*, 2015). Thus, the *COLD1* sequence found in tolerant *japonica* cultivars represents an ancient allele from *O. rufipogon* that was selected during domestication (Ma *et al.*, 2015). However, it is important to note that the SNP in *COLD1* explains only part of the cold tolerance in rice, a trait for which many minor effect QTLs are expected to contribute (Cruz *et al.*, 2013; Ma *et al.*, 2015; Mao *et al.*, 2015). Thus, other QTLs should be characterized and used in combination in order to develop highly tolerant lines, and newly identified genes from wild species might also be useful.

## Drought

Drought tolerance is a complex trait, with many genes and processes involved (Singh *et al.*, 2015). Rice in particular demands great amounts of water for proper development, owing to its shallow roots compared to other crops (Kondo *et al.*, 2000). QTLs for drought tolerance were identified within *O. sativa* variability, and causative genes have been cloned (Vikram *et al.*, 2011; Uga *et al.*, 2013). DRO1 (DEEPER ROOTING 1), a previously unknown protein, is responsible for downward growth of rice roots, and introgression of *DRO1* in otherwise shallow root rice genotypes increases root angle and drought tolerance (Uga *et al.*, 2013). Still, wild rice species might be a source of stronger drought tolerance genes and mechanisms, since they are adapted to a much wider spectrum of environments (Vaughan *et al.*, 2003; Atwell *et al.*, 2014).

Species that are present in low moisture regions were suggested as more likely candidates for drought tolerance, namely: *O. barthii, O. australiensis, O. glaberrima, O. longistaminata* and *O. punctata* (Atwell *et al.*, 2014). At least three of these (*O. australiensis, glaberrima* and *longistaminata*), plus *O. meridionalis*, present thick leaves and high mesophyll conductance to CO_2_ diffusion, indicating that they might be drought tolerant, since these traits can be associated with a higher water use efficiency (Scafaro *et al.*, 2011; Giuliani *et al.*, 2013). Indeed, field evaluation of *O. glaberrima* showed that some accessions could be used as donors in crossing with *O. sativa* for drought tolerance breeding (Ndjiondjop *et al.*, 2010).

The *O. rufipogon* genotype Dongxiang was also used to breed drought tolerance in *O. sativa*. An introgressed line was shown to be more tolerant when compared to the *O. sativa* recurrent parent, with higher survival rate, along with higher proline and soluble sugar accumulation (Zhang *et al.*, 2014). However, it is clear that the trait is genotype-specific, since different *O. rufipogon* accessions can have widely different sensitivity levels. Feng *et al.* (2012) tested tolerance of eight accessions of *O. rufipogon* and one of *O. officinalis*, and observed that accessions from tropical origin are more tolerant. Interestingly, the single *O. officinalis* (CC genome; Table 2) accession performed even better under drought conditions (Feng *et al.*, 2012).

Another study analyzed leaf rolling score and relative water content in several *O. sativa, O. rufipogon* and *O. nivara* genotypes from India, and associated these traits with sequence diversity of *OsDREB1F*, a known drought stress-responsive transcription factor (Wang *et al.*, 2008; Singh *et al.*, 2015). At least five truncated versions of *OsDREB1F* were found to be associated with drought sensitivity. Interestingly, one protein variant, present in four *O. nivara* accessions, was associated with high relative water content and low leaf rolling score. The variant harbors an amino acid mutation in a putative activation domain, which, based on molecular modeling, is likely to affect the tertiary structure of the protein (Singh *et al.*, 2015). The over-expression of *OsDREB1F* in *O. sativa* confers tolerance to drought, low temperature and salt stresses (Wang *et al.*, 2008). Thus, the protein variant from *O. nivara* genotypes is likely to confer tolerance to multiple stresses, possible due to effects on tolerance to the osmotic adjustment component that is common in these conditions.

## Heat

Rice is a pan-tropical species, and thus well adapted to high temperatures compared to other grasses. However, the increasing global average temperatures and the more frequent occurrence of heat waves (IPCC, 2007) could affect rice growth, both during vegetative and reproductive stages. Species from the *Oryza* genus were suggested as heat tolerant (Atwell *et al.*, 2014), but few have been physiologically characterized.

*O. meridionalis* (AA genome, Table 1), is endemic to hot regions of northern Australia, and is described as heat tolerant (Scafaro *et al.*, 2010). When compared to *O. sativa, O. meridionalis* leaf elongation rate is slower under 27 °C, but faster at 45 °C. The photosynthesis temperature optimum of *O. meridionalis* is 3 °C above that of *O. sativa*, which is accounted for by a higher RuBisCO activation state under heat stress (Scafaro *et al.*, 2012). Proteomics analyses showed that Calvin Cycle and heat shock-related proteins increased their abundance in *O. meridionalis* leaves at high temperatures, including Rubisco activase (Scafaro *et al.*, 2010). Thus, higher Rubisco activase accumulation is directly involved in heat tolerance of *O. meridionalis*, maintaining RuBisCO carboxylation at higher temperatures (Scafaro *et al.*, 2010, 2012).

Temperatures higher than 32-36 °C at anthesis cause spikelet sterility and yield reduction, mainly due to reduced anther dehiscence and germinating pollen on the stigma (Ishimaru *et al.*, 2010, and references therein). Flowering in cultivated rice occurs between mid-morning to noon, when heat has already built up since the beginning of the day (Nishiyama and Blanco, 1980). A useful trait to avoid flowering at high temperatures is early morning flowering (EMF). The wild rice species *O. officinalis* shows EMF, which can be used to increase cultivated rice fertilization and yield by escaping heat stress (Ishimaru *et al.*, 2010). Indeed, introgression lines were produced from *O. sativa* X *O. officinalis* crosses, and these showed increased fertility due to the shift in anthesis timing (Ishimaru *et al.*, 2010). QTL analyses revealed that an *O. officinalis* EMF candidate gene is located in chromosome 3 and reduces the flowering opening time by 1.5 to 2 hours in both temperate and tropical cultivars, thus demonstrating the usefulness of this trait to reduce effects of heat stress on spikelet sterility (Hirabayashi *et al.*, 2015).

## Concluding remarks

Early domestication of crops was a bottleneck for gene diversity. While selecting important traits for cultivation, the process inadvertently lost others that might be interesting for humans today. As an example, a wheat NAC transcription factor, NAM-B1, was shown to regulate iron, zinc and protein levels in grains. Interestingly, the NAM-B1 allele from wild emmer wheat is functional, accelerating senescence and increasing remobilization of nutrients to developing seeds, while the modern varieties carry a non-functional allele, and thus have decreased levels of iron, zinc and protein (Uauy *et al.*, 2006). Considering that iron and zinc deficiencies in humans are a common dietary problem (Sperotto *et al.*, 2012; Ricachenevsky *et al.*, 2015), introgression of the functional NAM-B1 allele is likely to improve wheat nutritional quality.

The use of wild relatives as a reservoir of new alleles to confer stress tolerance to cultivated species has been successful before. In wheat, introgression of the *Nax2* locus (containing the *TmHKT1;5-A* allele) from *Triticum monoccocum* conferred improved salt tolerance, with reduced Na^+^ accumulation in leaves and a 25% increase in grain yield in high salt soils, and resulted in a wide distribution of *Nax2* bearing germplasm to producers (James *et al.*, 2012; Munns *et al.*, 2012; Mickelbart *et al.*, 2015). A soybean wild relative, *Glycine soja*, has also been shown to be salt tolerant (Chen P *et al.*, 2013). A Cation H^+^ Exchange (CHX) transporter was identified as the causative gene of the tolerance phenotype, and gain-of-function transgenic plants expressing the transporter were shown to be more salt tolerant (Qi *et al.*, 2014).

Considering the wide variety of environments in which species from the *Oryza* genus are found, it is expected that they also vary in abiotic stress tolerance (Atwell *et al.*, 2014). Thus, screening for stress tolerance in these species, and within multiple genotypes of each species, should yield new alternatives for rice improvement. Efforts such as the International Oryza Map Alignment Project and the genome sequencing of 16 species (Jacquemin *et al.*, 2013) should fast track the unlocking of rice wild relatives potential for breeding and transgenic approaches. The combination of these data with large scale screening for tolerance in wild genotypes, together with the available and newly generated QTL maps and genome re-sequencing of tolerant individuals will be key to guarantee food security, considering the prospects of climate change and overpopulation.
